# Homeostatic Proliferation Fails to Efficiently Reactivate HIV-1 Latently Infected Central Memory CD4+ T Cells

**DOI:** 10.1371/journal.ppat.1002288

**Published:** 2011-10-06

**Authors:** Alberto Bosque, Marylinda Famiglietti, Andrew S. Weyrich, Claudia Goulston, Vicente Planelles

**Affiliations:** 1 Department of Pathology, University of Utah, Salt Lake City, Utah, United States of America; 2 International PhD School in Molecular Medicine, Basic and Applied Immunology Section, Vita-Salute San Raffaele University, Milano, Italy; 3 Program in Human Molecular Biology and Genetics, Department of Internal Medicine, University of Utah School of Medicine, Salt Lake City, Utah, United States of America; 4 Division of Infectious Diseases, Department of Medicine, University of Utah, Salt Lake City, Utah, United States of America; Fred Hutchinson Cancer Research Center, United States of America

## Abstract

Homeostatic proliferation ensures the longevity of central memory T-cells by inducing cell proliferation in the absence of cellular differentiation or activation. This process is governed mainly by IL-7. Central memory T-cells can also be stimulated via engagement of the T-cell receptor, leading to cell proliferation but also activation and differentiation. Using an *in vitro* model of HIV-1 latency, we have examined in detail the effects of homeostatic proliferation on latently infected central memory T cells. We have also used antigenic stimulation via anti-CD3/anti-CD28 antibodies and established a comparison with a homeostatic proliferation stimulus, to evaluate potential differences in how either treatment affects the dynamics of latent virus populations. First, we show that homeostatic proliferation, as induced by a combination of IL-2 plus IL-7, leads to partial reactivation of latent HIV-1 but is unable to reduce the size of the reservoir in vitro. Second, latently infected cells are able to homeostatically proliferate in the absence of viral reactivation or cell differentiation. These results indicate that IL-2 plus IL-7 may induce a detrimental effect by favoring the maintenance of the latent HIV-1 reservoir. On the other hand, antigenic stimulation efficiently reactivated latent HIV-1 in cultured central memory cells and led to depletion of the latently infected cells via virus-induced cell death.

## Author Summary

HIV-1 latently infected cells are considered the last barrier towards viral eradication and cure. However, the low number of latently infected cells found in patients makes studies extremely difficult. Here, using a model of primary CD4 T-cells we study the behavior of latently infected central memory T cells when undergoing homeostatic proliferation. Homeostatic proliferation ensures the longevity of the central memory population, as it does not involve cellular differentiation. In the context of HIV infection, IL-7 has been reported to induce viral outgrowth from latently infected cells in different cellular models. However, those studies did not examine the relationship between cell proliferation and viral reactivation. We here report that the strong effect of IL-7 on the proliferation of memory cells counteracts this cytokine's modest ability to purge latent viruses. Thus, central memory cells are subject to homeostatic proliferation, a physiological effect that may contribute to the longevity of the latent reservoir in HIV-1 infected patients.

## Introduction

The existence of latent reservoirs of HIV-infected cells constitutes a major impediment to viral eradication. HIV-1 latent reservoirs are small, but extremely long-lived. Latent infection is associated with undetectable levels of viral gene expression and appears to be non-cytopathic. However, upon reactivation, latent viruses enter an active mode of replication in which they are fully competent for spread and induction of disease [1], [2], [3]. It is unclear which physiological stimuli may trigger or prevent viral reactivation in latently infected cells. Obvious possibilities include antigenic stimulation, inflammatory conditions, and, perhaps, certain immunological microenvironments. Regarding potential therapies, the current thinking in the field is that a combination of hypothetical drugs that will reactivate latent viruses (“anti-latency” drugs), with present-day antiretroviral drugs, will be an effective approach toward viral eradication [1], [4], [5]. However, we are limited by the lack of known drugs that can safely be used to induce viral reactivation in patients. We are also limited by our poor understanding of how cellular and viral factors govern the establishment of latency and the reactivation process.

Memory is a hallmark of the acquired immune system and results from the clonal expansion and differentiation of antigen-specific lymphocytes that persist for a lifetime. Memory T cells result from the activation and differentiation of naïve T cells and perform two indispensable and complementary functions, which are carried out by different cellular subsets [6]. Effector memory T cells (T_EM_) migrate to inflamed peripheral tissues and display immediate effector function. On the other hand, central memory T cells (T_CM_) home to areas of secondary lymphoid organs where, in response to antigenic stimulation, they can vigorously proliferate and differentiate to T_EM_. In the case of the CD4+ memory T cells, the effector subset is further subdivided into several T-helper types, such as T_H_1, T_H_2 and T_H_17, among others, which are characterized by the expression of specific chemokine receptors and the production of specific cytokines like IFNγ, IL-4 or IL-17, respectively [7].

The proliferation of memory T cells can be driven by antigenic stimulation (antigen-driven proliferation) or by cytokines (homeostatic proliferation). Through homeostatic proliferation, the immune system is able to maintain normal T-cell counts, and to correct for deviations due to expansion or depletion of the memory cell pool [8], [9], [10]. Homeostatic proliferation is governed by extrinsic cellular signals, typically γc-cytokines, in the absence of antigenic stimulation. For CD4^+^ memory T cells, IL-7 is key in governing homeostasis [8], [11], [12]. A role for IL-15, another γc-cytokine, has also been proposed [11].

Previous work by several groups (reviewed in [13], [14]) suggested that *in vivo*, quiescent memory T-cells constitute a long-lived viral reservoir, whose decay constant ranges from months to years. A recent report has provided additional evidence to support the role of central memory T cells (T_CM_), along with transitional memory T cells, as the main latent viral reservoirs *in vivo* [15]. The Chomont et al. study also proposed that cells in the latent reservoir may be able to undergo homeostatic proliferation (as evidenced by the presence of the Ki67 marker), and this in turn would provide a means for the maintenance or even expansion of the HIV-1 latent reservoir [15]. While this idea could not be tested in patient cells due to the low abundance of latent infection, we were able to test the effect of homeostatic proliferation of latently infected cells in our cultured T_CM_ model of latency [16]. Using this model, we confirm the ability of T_CM_ cells to homeostatically proliferate in response to IL-2 plus IL-7, in a manner that is indistinguishable between uninfected and latently infected ones. We also demonstrate that proliferation of infected cells in response to IL-2 plus IL-7 stimulation is accompanied by inefficient viral reactivation. Because of the inefficient viral reactivation, many residual latently infected cells remain which can later be reactivated using antigenic stimulation.

## Results

### Antigenic stimulation of latently infected cultured T_CM_ effectively depletes the latent reservoir whereas homeostatic proliferation fails to do so

**Figure 1:**
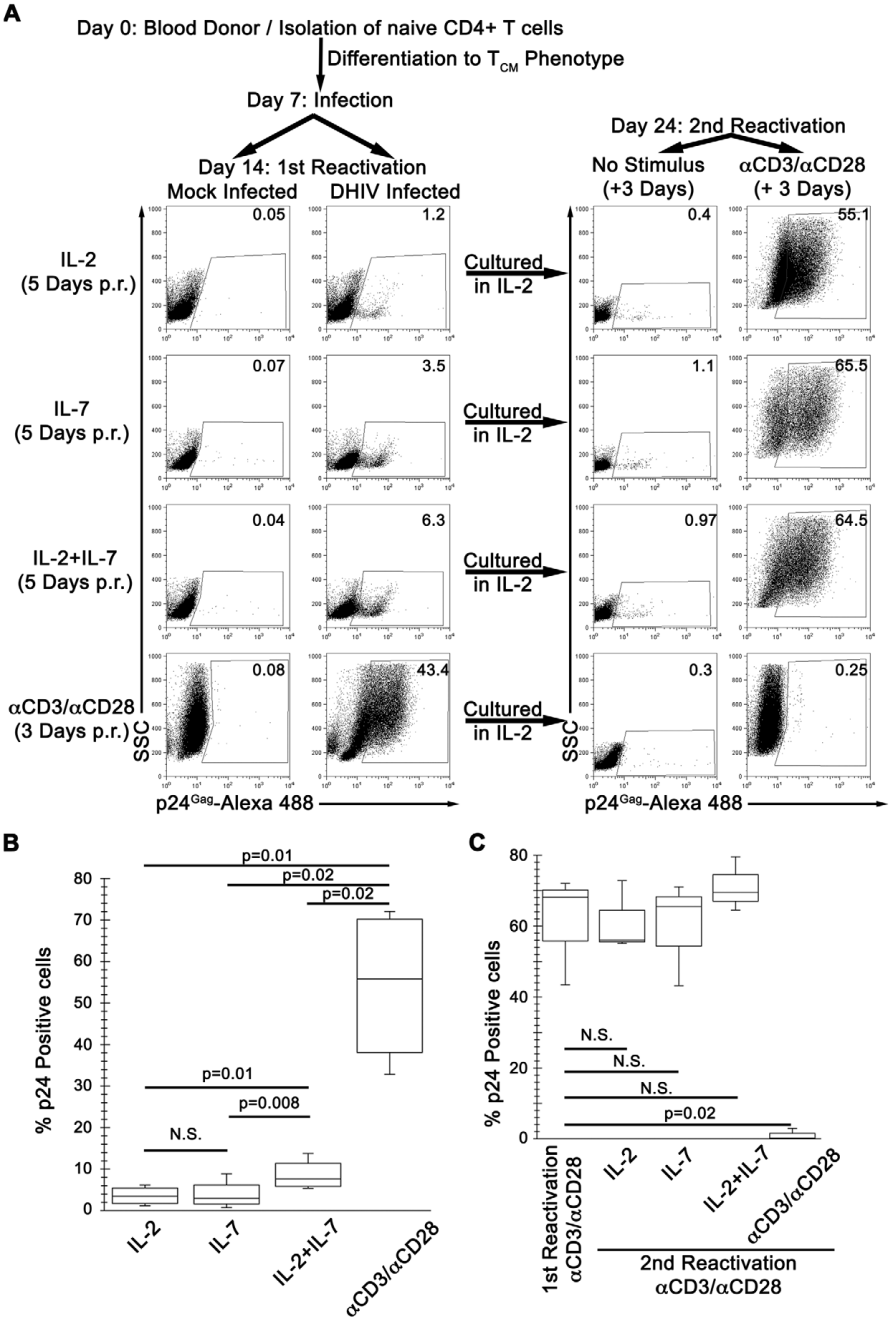
IL-7 induces partial reactivation of latent HIV-1 in cultured T_CM_. (A) Mock or DHIV latently infected, cultured T_CM_ were incubated in the presence of IL-2, IL-7 or a combination of IL-2 and IL-7 (IL-2+IL-7) or costimulated with antibodies to CD3 and CD28 (αCD3/αCD28) and assessed for intracellular p24Gag by flow cytometry (1st Reactivation). The percentage of p24Gag positive cells is indicated in each panel. After reactivation, cells were kept in culture for an additional 7-day period in the presence of IL-2. At day 24, cells were incubated in the presence of IL-2 or costimulated with antibodies to CD3 and CD28 (αCD3/αCD28) and assessed for intracellular p24Gag by flow cytometry (2nd Reactivation). Data is representative of 4 donors for 1^st^ reactivation and 3 donors for 2^nd^ reactivation (p.r. post reactivation). (B) Box-plots corresponding to the 4 donors analyzed as in A, left panels. Horizontal lines indicate median values; statistical significance was assessed by 2-tailed paired-sample *t* test analysis (P values provided, N.S. not significant). (C) 3 of these previous donors were analyzed as in A (right panels, 2nd Reactivation), and compared with the first reactivation with αCD3/αCD28. Horizontal lines indicate median values; statistical significance was assessed by 2-tailed paired-sample *t* test analysis (P values provided, N.S. not significant).

To investigate the effect of homeostatic proliferation and antigen stimulation signals on the dynamics of a latent infection, we first established a population of latently infected cultured, non-polarized (NP) cells, as previously described [16]. NP cells are considered the *in vitro* equivalent of *in vivo* T_CM_ [17], [18] and we will hereinafter refer to them as “*cultured*” T_CM_. Peripheral blood mononuclear cells (PBMC) were obtained from healthy human donors, and naïve CD4+ cells were isolated via negative selection [18]. Purified naïve CD4^+^ T cells were activated (day 0 to day 3) by incubation with beads coated with αCD3 and αCD28 antibodies in medium containing TGF-β1, αIL-12 and αIL-4 antibodies, and then cultured for 4 additional days with IL-2 (Figure 1A). Cells were then infected with 500 ng of p24 of the virus, DHIV/X4. DHIV/X4 is an envelope-defective molecular clone derived from HIV-1_NL4-3_, which is pseudotyped with the HIV-1_LAI_ envelope glycoprotein. Because of the *env* deletion, this virus is only capable of a single round of infection.

After infection, cells were cultured in medium containing IL-2 for an additional 7 days. Under these conditions, most of the cells returned to a quiescent state characterized as G_0_ as evidenced by low pyronin and 7-AAD staining intensities (Figure S1), where the vast majority of proviruses exist in a latent state [16]. The continuous presence of IL-2 in the culture did not interfere with either the establishment of latency or the return to quiescence of the cells [16]. IL-2 was, however, required for survival of the cells in culture (data not shown). At day 14 (first reactivation; Figure 1A), we tested the ability of IL-7 to induce viral reactivation in cultured T_CM_ cells. To that end, cells were exposed to normal medium containing IL-2, IL-7 or a combination of IL-2 plus IL-7 (IL-2+IL-7). As a positive control for virus reactivation, cells were incubated with beads coated with αCD3/αCD28 antibodies. In a representative donor shown in Figure 1A, latently infected cells stimulated with αCD3/αCD28 displayed 43.4% p24Gag^+^ cells, while incubation with IL-2+IL-7 only resulted in 6.3% p24Gag^+^ cells. Treatments with IL-2 or IL-7 alone reactivated latent viruses even less efficiently (1.2 and 3.5%, respectively). When we performed similar studies with cells from three additional healthy donors, IL-7 alone failed to reactivate latent HIV-1 in two donors. However, IL-2+IL-7 treatment reactivated latent HIV-1 in all four donors with a frequency that was, on average, one tenth of that obtained with αCD3/αCD28 incubation (Figure 1B). Therefore, IL-7 treatment reactivated latent viruses in 2 out of 4 donors and, when used in combination with IL-2, in 4 out of 4 donors; and always with a lower efficiency than that obtained with antigenic stimulation. Our results are in agreement with published reports showing that incubation with IL-7, either alone [19] or in combination with IL-2 [20] can reactivate latent HIV-1 in resting CD4+ T cells isolated from infected individuals. IL-7 also reactivated latent HIV-1 in thymocytes in a SCID-hu mouse model of HIV latency [21].

To explore the relatively low efficiency of viral reactivation induced by IL-7 alone or in combination with IL-2 we measured the levels of expression of the high-affinity alpha chain receptors for IL-2 or IL-7 (CD25 and CD127, respectively). As shown in Figure S2A, the frequencies of cultured T_CM_ expressing CD25 and CD127 were high (81% and 24%, respectively), and similar to those observed in freshly isolated T_CM_ (66% and 31%, respectively) although the mean fluorescence intensities (MFI) were low. For CD25, when cells were stimulated with αCD3/αCD28, MFI increased by almost two orders of magnitude (Figure S2B). In addition, a dose-response study indicated that the levels of IL-7 we utilized in the experiment shown in Figure 1 (50 ng/ml) and throughout the rest of the study produced near-maximal response in these cells (Figure S2C). Therefore, the low relative efficiency of viral reactivation induced by IL-2+IL-7 was also not due to suboptimal amounts of IL-7.

A minority of cells in the cytokine treatments shown in Figure 1A reactivated latent viruses as evidenced by intracellular p24 expression. The remainder of the cells failed to reactivate latent proviruses and, therefore, we predicted that they remained latently infected. To prove that residual latent viruses were indeed present after cytokine stimulation, we cultured these cells in IL-2 for an additional week, and then proceeded to stimulate with αCD3/αCD28 antibodies (day 24, second reactivation; Figure 1A, right panels). The latently infected culture that had previously been reactivated with αCD3/αCD28 antibodies (first reactivation) and produced 43.4% p24Gag^+^ cells, failed to produce additional p24Gag^+^ cells (0.25%) upon the second reactivation (Figure 1A, αCD3/αCD28 right panel).

The reasons for the lack of p24Gag^+^ cells after the second reactivation with αCD3/αCD28 are as follows. First, αCD3/αCD28 treatment induces reactivation of virtually all latently infected cells in the cultured T_CM_ model [16]. Second, cells in which viruses are reactivated undergo cell death [16], [22]. Third, DHIV is a defective virus, which is unable to spread after the initial infection. Therefore, new infections are not generated when latent viruses reactivate. This simplifies the analysis because it rules out virus spread as a potential confounding factor upon reactivation. It also obviates the use of anti-retrovirals in the cell cultures in order to suppress viral spread. To ascertain whether, in this *in vitro* system, αCD3/αCD28 stimulation effectively depletes the latent reservoir, we performed a parallel experiment in which we also measured integrated HIV-1 DNA by Alu-LTR PCR prior to the first and the second reactivations. As shown in Figure S3B, the levels of integrated DNA in DHIV infected cells prior to the second reactivation decreased to background, undetectable levels, confirming the absence of residual latently infected cells.

Latently infected cells that had been exposed either to IL-2, IL-7, or IL-2+IL-7, when later exposed to αCD3/αCD28 antibodies, produced numerous p24Gag^+^ cells (55.1%, 65.5% and 64.5%, respectively, Figure 1B right panels). Therefore, latently infected cells that failed to reactivate in the presence of no stimulus (IL-2 alone) or a weak stimulus (IL-7 or IL-2+IL-7) remained as an inducible reservoir. Similar results were obtained in a total of three donors (Figure 1C).

### Reactivation of latent HIV-1 by IL-2+IL-7 in quiescent T_CM_ is independent of entry into the cell cycle

**Figure 2:**
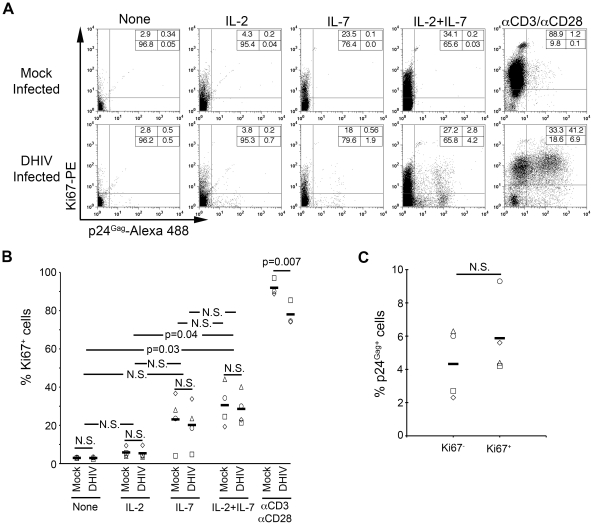
IL-7 induces cell cycle entry of cultured T_CM_. (A) Mock or DHIV latently infected, cultured T_CM_ were left in the absence of cytokines (None) or in the presence of IL-2, IL-7 or a combination of IL-2 and IL-7 (IL-2+IL-7) or costimulated with antibodies to CD3 and CD28 (αCD3/αCD28) and assessed for intracellular p24Gag and Ki67 expression by flow cytometry. Numbers in boxes indicate percentages. (B) Analysis corresponding to 4 different donors analyzed as shown in panel A; each donor is represented with a different symbol and horizontal lines indicate media values. Statistical significance was calculated by 2-tailed paired-sample *t* test analysis (P values provided, N.S. not significant). (C) Analysis of the percentage of p24Gag^+^ cells in each compartment (Ki67^−^ or Ki67^+^) after IL-2+IL-7 treatment in the four donors analyzed in B. Data was normalized as indicated in the text. Horizontal lines indicate media values. Statistical significance was calculated by 2-tailed paired-sample *t* test analysis (N.S. not significant).

Homeostatic proliferation of central memory T cells consists of entry into the cell division cycle, in the absence of cellular differentiation [12]. To confirm that IL-7 treatment alone, or in combination with IL-2, induces homeostatic proliferation, we determined the division status of the cells via staining for the nuclear antigen, Ki67. Ki67 is a nuclear marker that is present during all active phases of the cell cycle (G_1_, S, G_2_ and mitosis), but is absent in resting (G_0_) cells [23]. Ki67 staining was performed in conjunction with p24Gag staining, in order to evaluate whether viral reactivation was interdependent with entry into the cell cycle (Figure 2A). For the donor shown in Figure 2A, IL-2+IL7 incubation induced the strongest proliferation signal compared to other cytokine treatments or the no-cytokine control. The proportions of Ki67^+^ cells were similar in infected and mock-infected cultures (Figure 2A). Similar results were obtained in cells from four donors (Figure 2B). These observations indicate that the ability of cultured T_CM_ cells to enter cell division upon IL-2+IL-7 stimulation is not affected by the presence of latent infections.

It is important to note that IL-2+IL-7 stimulation induced latently infected cells to express p24Gag both in the dividing, Ki67^+^ (2.8%), and in the non-dividing, Ki67^−^ (4.2%) compartments (Figure 2A). We normalized the above frequencies of reactivation to the proportions of cells that were Ki67^+^ [2.8/(27.2+2.8)]×100 = 9.3% or Ki67^−^ [4.2/(65.8+4.2)]×100 = 6.0%. We performed this analysis for the four donors shown in Figure 2B. The results showed not statistically significant differences (n = 4; p = 0.29; Figure 2C). Thus, we conclude, that entry into the cell cycle does not influence, positively or negatively, whether a latent virus will be reactivated by treatment with IL-2+IL-7.

Analysis of the cell surface phenotype of the cytokine-treated cultures revealed that the majority of the cells maintained the phenotype of central memory cells, characterized by dual expression of CCR7 and CD27. Therefore, the IL-7 or IL-2+IL-7 induced cellular proliferation did not change the differentiation status of cultured T_CM_ cells, a hallmark of homeostatic proliferation (Figure S4).

### Latently infected, cultured T_CM_ undergo homeostatic proliferation in the absence of viral reactivation

The above experiments focused on the fraction of cells that reactivated virus under homeostatic proliferation, but did not reveal whether residual proviruses that remain latent after IL-7 or IL-2+IL-7 stimulation may be present within the proliferating (Ki67^+^) or non-proliferating (Ki67^−^) populations. Ki67 and p24Gag co-staining require fixation and permeabilization of the cells, which precludes further culture and reactivation of cells. Therefore, to more closely examine the fate of the latently infected population, we resorted to a different experimental scenario, as follows. To track cell division, we used Cell Proliferation Dye eFluor 670 (CPe670), a red-fluorescent dye that is analogous to the well-known carboxyfluorescein succinimidyl ester (CFSE). The progressive 2-fold dilution of CPe670 fluorescence within daughter cells following each cell division is used as an easy and quantitative means for measuring cell division in individual cells. The use of the red CPe670 allowed us to use a virus that expresses GFP in place of *nef* (DHIV-GFP). The combined use of CPe670 and GFP was designed to allow us to monitor and sort infected cells, assess cell division status, and continue to culture the cells thereafter.

We previously showed that GFP expression driven by DHIV-GFP closely parallels that of p24Gag and that the absence of *nef* did not modify the ability of HIV-1 to establish latent infection or to reactivate in cultured T_CM_ [16]. A latent infection was established using DHIV-GFP (day 7), and at day 14, cells were labeled with CPe670; cells were then treated with either IL-2 alone or IL-2+IL-7. We decided to use the combination of both cytokines and not IL-7 alone because the combination induced a stronger and more consistent proliferative effect among different donors (Figures 2A and 2B) without altering the phenotype of the cells (Figure S4).

**Figure 3:**
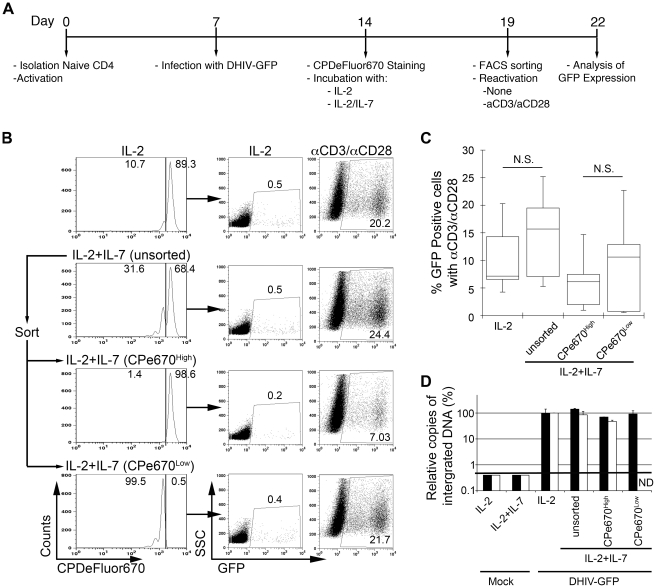
Most latently infected cells can proliferate in response to IL-2 plus IL-7 without inducing viral reactivation. (A) Timeline of the experiment. (B) At day 19, cells treated with IL-2 plus IL-7 (unsorted) were subjected to cell sorting into two populations: CPe670^High^ (undivided cells) or CPe670^Low^ (divided cells). The percentages of CPe670^High^ and CPe670^Low^ cells are indicated in the histograms. After sorting, cells were incubated in the presence of IL-2 or costimulated with antibodies to CD3 and CD28 (αCD3/αCD28) and assessed for GFP expression by flow cytometry. The three groups of cells treated with IL-2+IL-7 were compared with the same cells treated with IL-2 alone (IL-2). The percentages of GFP positive cells are indicated in each panel. (C) Box-plots corresponding to the 5 donors analyzed as in B. Horizontal lines indicate median values; statistical significance was assessed by 2-tailed paired-sample *t* test analysis (N.S. not significant). (D) Integrated HIV-1 DNA was analyzed by Alu-LTR PCR in duplicates in two donors from panel C. Results were normalized for each donor relative to the levels of integration in cells treated with IL-2. Black bars correspond to a donor with 6.5% of GFP positive cells after reactivation with αCD3/αCD28 of cells treated with IL-2. White bars correspond to a donor with 4.2% of GFP positive cells after reactivation with αCD3/αCD28 of cells treated with IL-2. Mock infected cells retrieved a value below the threshold of detection of the technique (ND not determined).

Five days after the above treatment (day 19; Figure 3A), cells were sorted by flow cytometry in order to (a) remove the GFP^+^ fraction; and (b) separate the GFP^−^ fraction into CPe670^High^ (having undergone no cell divisions) or CPe670^Low^ (having undergone one or more cell divisions). As shown in Figure 3B, only 10.7% of the IL-2-cultured cells underwent cell division, whereas 31.6% of the cells did so when incubated with IL-2+IL-7. After sorting, the four cultures shown in Figure 3B (IL-2, IL-2+IL-7 [unsorted], IL-2+IL-7 [CPe670^High^] and IL-2+IL-7 [CPe670^Low^]) were placed back in culture and reactivated with αCD3/αCD28 to reactivate latent viruses; or they were cultured in IL-2 for 3 days. Cells were then analyzed for GFP expression. As shown in Figure 3B (right panels), all four cultures were able to produce GFP^+^ cells after αCD3/αCD28 reactivation. The frequencies of GFP^+^ cells induced by the αCD3/αCD28 treatment correspond to the frequencies of latency prior to reactivation. The IL-2+IL-7 stimulated, unsorted culture revealed 24.4% GFP^+^ cells after αCD3/αCD28 reactivation. Cells that underwent no divisions (CPe670^High^) sorted from the previous culture produced 7.03% GFP^+^ events, whereas those that had divided at least once (CPe670^Low^) produced 21.7% GFP^+^. Therefore, we conclude that cells that fail to reactivate latent HIV-1 in response to homeostatic proliferation signals manage to preserve latent infections, and this occurs whether cells divide or not.

The results from the above analysis was extended to a total of 5 different donors, and the results are summarized in Figure 3C. The frequencies of latent proviruses present in the dividing versus the non-dividing cell populations are not statistically significant (n = 5; p = 0.36). To directly determine the levels of latently infected cells in these subsets in a manner that does not relay on viral reactivation, Alu-LTR PCR was performed in cells from two donors (white and black bars in Figure 3D) after the cell sorting. As shown in Figure 3D, the levels of integrated DNA remained similar in cells treated with IL-2 or IL-2+IL-7; and between cells that had divided (CPe670^High^) versus those that had not (CPe670^Low^). Therefore, we conclude that latently infected cells can proliferate in response to homeostatic proliferation signals, allowing for mitotic spread of the latent proviruses within.

## Discussion

Previous studies have shown that IL-7 or IL-2+IL-7 stimulation is able to induce HIV-1 reactivation in latently infected, resting cells isolated from infected individuals [19], [20] and in thymocytes from a SCID-hu mouse model of HIV-1 latency [21]. However, the above studies did not analyze the effect of homeostatic proliferation on the size of the latent reservoir in T_CM_. In the present study, we show that, although homeostatic proliferation signals are able to induce partial viral reactivation from HIV-1 latency in T_CM_ cells, cell proliferation may nullify the potential benefits of IL-7 as a natural “anti-latency” treatment. It is noteworthy that while incubation with IL-2 alone induced very low levels of reactivation in our system, others have reported significant effects of IL-2 [24]. Two crucial differences come to mind when trying to reconcile our study with that of Gondois-Rey et al. [24]. First, Gondois-Rey et al. used PHA instead of αCD3/αCD28 for T-cell stimulation. Second, our studies isolate cells with a pure central memory phenotype, whereas Gondois-Rey et al. did not discriminate between central, transitional and effector memory cells. These discrepancies may underlie dramatic differences in the responsiveness of various T cell subsets to IL-2 and should be further examined.

Because HIV-1 latently infected T_CM_ cells do not detectably express viral antigens, there is no reason to suspect that homeostatic proliferation will occur at a different rate in infected versus uninfected cells. In fact, our results using cultured T_CM_ indicate that there is not a statistically significant difference between latently infected cells and uninfected ones (Figures 3C) in their abilities to proliferate. We surmise that the normal ability of latently infected cells to proliferate is an important contributor toward their long-term maintenance. This result also indicates that the resting state is not an absolute requirement toward establishing latency. A clear precedent for this notion is the well known fact that certain immortalized cell lines can harbor latent viruses indefinitely, which are capable of being reactivated [25], [26]. Therefore, cell division status can be independent of latency, and we here extended this notion to primary T_CM_ cells.

Using cultured T_CM_ cells, we were also able to model the dramatic effect of antigenic stimulation on the size of the latent reservoir in T_CM_. The results indicate that antigenic stimulation can deplete the latent reservoir via viral reactivation, whereas weak stimuli, such as IL-2 or IL-2+IL-7, are unable to do so (Figure 1).

In this report, we carefully analyze the effect of homeostatic proliferation and antigen stimulation on HIV-1 latently infected T_CM_ *in vitro*. As this reservoir *in vivo* is extremely long-lived and impervious to conventional anti-retroviral treatment, analysis of other factors that may impact the latent reservoir in patients is extremely compelling. Such factors will likely include a plethora of immunological mediators, general and specific inflammatory conditions, and the presence of other infectious agents.

In addition to the documented ability of IL-7 to induce reactivation of latent HIV-1 in resting cells from patients [19], [20], recombinant IL-7 (rIL-7) has shown efficacy in combating lymphopenia when administered to HIV-1 patients [27]. These benefits, combined with the low toxicity observed in the setting of rIL-7 administration, have prompted several clinical trials with the goal of eradicating the HIV-1 reservoir with rIL-7 administration. In the past, potential anti-latency drugs were evaluated for their ability to induce virus replication in the face of minimal cellular activation [28], [29]. Given the results presented here, we propose that potential “anti-latency” drugs should also be examined for the undesired ability to induce cellular proliferation in the presence of incomplete viral reactivation.

## Methods

### Reagents

The following reagents were obtained through the AIDS Research and Reference Reagent Program, Division of AIDS, NIAID: Human rIL-2 from Dr. Maurice Gately, Hoffman-La Roche Inc. [30]; and Monoclonal Antibody to HIV-1 p24 (AG3.0) from Dr. Jonathan Allan [31].

### Generation of cultured T_CM_ cells and their latent infection

Peripheral blood mononuclear cells were obtained from Leukopaks from unidentified, healthy donors. Cultured T_CM_ and latently infected cultured T_CM_ were generated as previously described [16], [18].

### Ethics statement

Cells from uninfected blood donors. Human subjects 18 years and older serve as blood donors. Written informed consent was obtained from all donors. These studies are covered under the IRB #392 protocol approved by the University of Utah Institutional Review Board. The amount of blood drawn varies, depending on the experiments to be done. This ranges from as little as 50 milliliters (ml) to as much as 500 ml in young, healthy donors.

### Reactivation assays

2.5×10^5^ cells were reactivated with beads coated with αCD3 and αCD28 (1 bead per cell, Dynal/Invitrogen, Carlsbad, CA) during 3 days in the presence of 30 IU/ml IL-2; with IL-2 alone; with 50 ng/ml of IL-7 alone or with a combination of IL-2 and IL-7 (Peprotech, Rocky Hill, NJ) for 5 days.

### Flow cytometry

To assess intracellular p24Gag expression, 2.5×10^5^ cells were fixed, permeabilized, and stained as previously described [16].

To phenotype the cells, 2.5×10^5^ cells were stained with surface marker-specific mAb specific for human: PE-anti-CD25 (Caltag, Burlingame, CA), FITC-anti-CD127 (eBioscience, San Diego, CA), PE-anti-CD27 (Caltag, Burlingame, CA) or FITC-anti-CCR7 (R&D Systems, Minneapolis, MN) followed by flow cytometric analysis in a BD Facscanto II flow cytometer using the FACSDiva software (Becton Dickinson, Mountain View, CA) and analyzed using FlowJo (Tree Star Inc., Ashland, OR). To analyze the expression of CD25 and CD127 in *ex vivo* isolated T_CM_, memory CD4+ T cells were isolated by MACS microbead-negative sorting using the memory CD4+ T cell isolation kit (Miltenyi Biotec, Auburn, CA). After sorting, cells were stained with FITC-anti-CD127, PE-anti-CD27 and APC-anti-CCR7 (R&D Systems, Minneapolis, MN); or FITC-anti-CD27 (Caltag, Burlingame, CA), PE-annti-CD25 and APC-anti-CCR7 followed by flow cytometric analysis as described above.

To analyze the dual expression of Ki67 and p24Gag, 5×10^5^ cells were fixed, permeabilized and stained for p24Gag as previously described [16]. After p24Gag staining, cells were washed with Perm/Wash Buffer (BD Biosciences) and were incubated with 1/100 dilution of Mouse IgG1,κ (MOPC-21, Sigma, Saint Louis, MO) in 100 µl of Perm/Wash Buffer for 30 min at 4°C to remove excess of secondary antibody. Cells were then washed with Perm/Wash Buffer and incubated with a 1/20 dilution of anti-Ki67-PE (BD Biosciences) in 100 µl of Perm/Wash Buffer for 30 min at 4°C. Cells were washed with Perm/Wash Buffer and samples were analyzed on a FACSCalibur flow cytometer. Forward versus side scatter profiles were used to define the live population. In all the experiments, HIV p24Gag negative staining regions were set with uninfected cells treated in parallel and Ki67 negative staining regions were set with the corresponding IgG-PE.

To analyze cell division with Cell Proliferation Dye eFluor 670 (eBioscience, San Diego, CA), cells were stained as indicated by the manufacturer.

To analyze DNA and RNA content, samples were stained with 7-aminoactinomycin D (7-AAD) and pyronin Y (PY) as previously described [32].

### Integration analysis

Genomic DNA was isolated with DNeasy Tissue Kit (QIAGEN, Valencia, CA). Genomic DNA (250 ng) was subjected to quantitative Alu-LTR polymerase chain reaction (PCR) for integrated proviruses as previously described [33], [34].

## Supporting Information

Figure S1 (A) Analysis of DNA (7-AAD) and RNA (PY) content in cultured T_CM_. Cells were stained with 7-AAD and PY at the time of reactivation (Day 14) in the presence of IL-2 (IL-2) or in the absence of IL-2 for 4 days (None). Numbers in boxes indicate percentages. The different phases of the cell cycle are indicated in the right panel.
(TIF)

Figure S2 (A) Analysis of expression of CD25 (IL-2Rα) and CD127 (IL-7Rα) in cultured T_CM_ or *ex vivo* T_CM_. The percentage of positive cells is indicated. (B) Analysis of expression of CD25 (IL-2Rα) in cultured T_CM_ (Black-filled histogram) or cultured T_CM_ activated with aCD3/aCD28 antibodies for 72 hours (Dark-filled histogram). IgG isotype control is the light grey-filled histogram. (C) Dose-response curve of viral reactivation induce by a combination of IL-2 at 30 IU/ml and increasing concentrations of IL-7. The percentage of p24Gag positive cells is indicated in the Y-axis.
(TIF)

Figure S3 (A) Levels of p24Gag positive cells after reactivation with αCD3/αCD28 antibodies in the 1^st^ and 2^nd^ reactivation for a representative donor. (B) Integrated HIV-1 DNA was analyzed by Alu-LTR PCR in triplicates in the donor from panel A prior to reactivation. Results were normalized relative to the levels of integration in DHIV infected cells before the first reactivation. Mock infected cells and DHIV infected cells prior to second reactivation retrieved a value below the threshold of detection of the technique (C) Amplification curves form the same samples used for B.
(TIF)

Figure S4 Expression of CCR7 and CD27 in mock or DHIV-latently infected cultured T_CM_ cells at day 14 (Day 7 p.i.). The expression was also monitored 5 days after incubation of the cells in the absence of cytokines (none) or with IL-2, IL-7 or a combination of IL-2 and IL-7 (IL-2+IL-7).
(TIF)
