# Supplementary figures and images for: Homeostatic Proliferation Fails to Efficiently Reactivate HIV-1 Latently Infected Central Memory CD4+ T Cells
Source: PLoS Pathog. 2011 Oct 6;7(10):e1002288. doi: 10.1371/journal.ppat.1002288 (PMC3188522; doi:10.1371/journal.ppat.1002288)

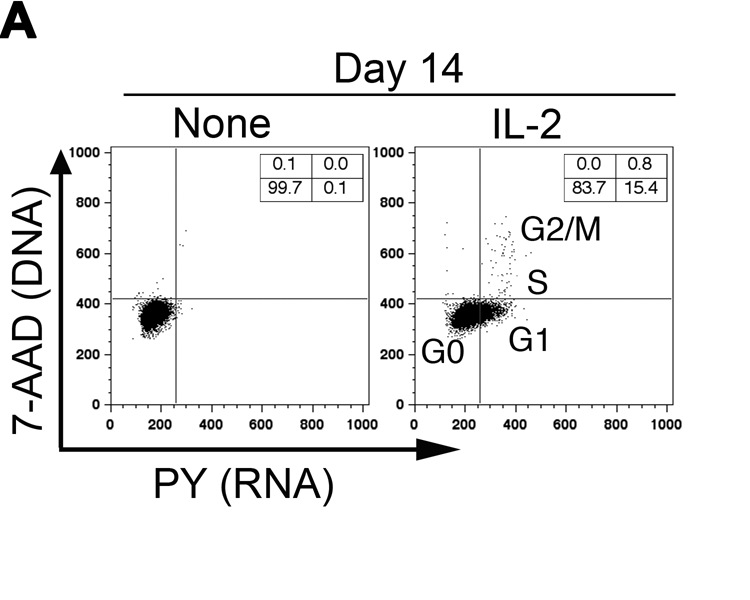

Supplement: Figure S1 — (A) Analysis of DNA (7-AAD) and RNA (PY) content in cultured TCM. Cells were stained with 7-AAD and PY at the time of reactivation (Day 14) in the presence of IL-2 (IL-2) or in the absence of IL-2 for 4 days (None). Numbers in boxes indicate percentages. The different phases of the cell cycle are indicated in the right panel. (TIF) [file ppat.1002288.s001.tif]

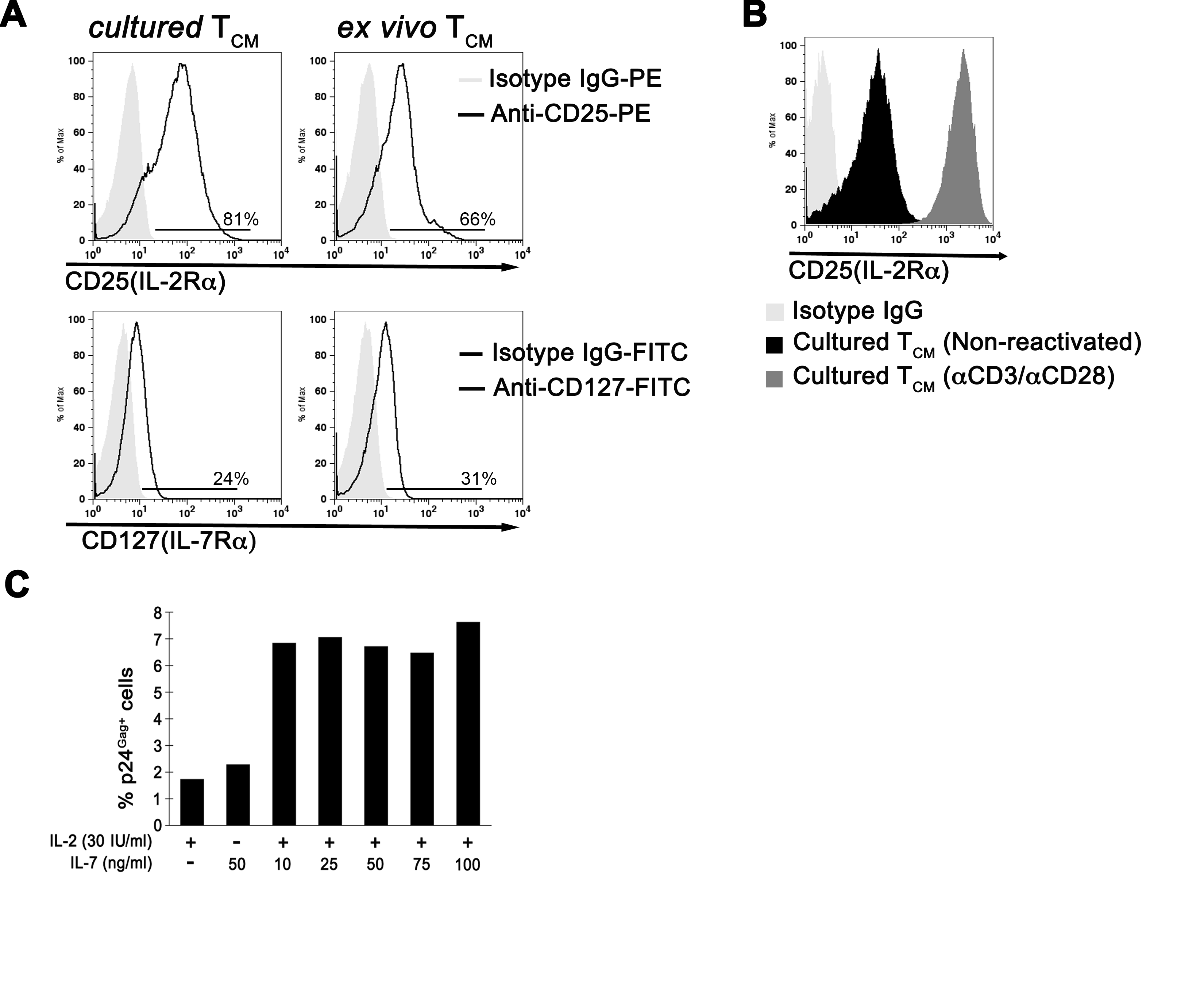

Supplement: Figure S2 — (A) Analysis of expression of CD25 (IL-2Rα) and CD127 (IL-7Rα) in cultured TCM or ex vivo TCM. The percentage of positive cells is indicated. (B) Analysis of expression of CD25 (IL-2Rα) in cultured TCM (Black-filled histogram) or cultured TCM activated with aCD3/aCD28 antibodies for 72 hours (Dark-filled histogram). IgG isotype control is the light grey-filled histogram. (C) Dose-response curve of viral reactivation induce by a combination of IL-2 at 30 IU/ml and increasing concentrations of IL-7. The percentage of p24Gag positive cells is indicated in the Y-axis. (TIF) [file ppat.1002288.s002.tif]

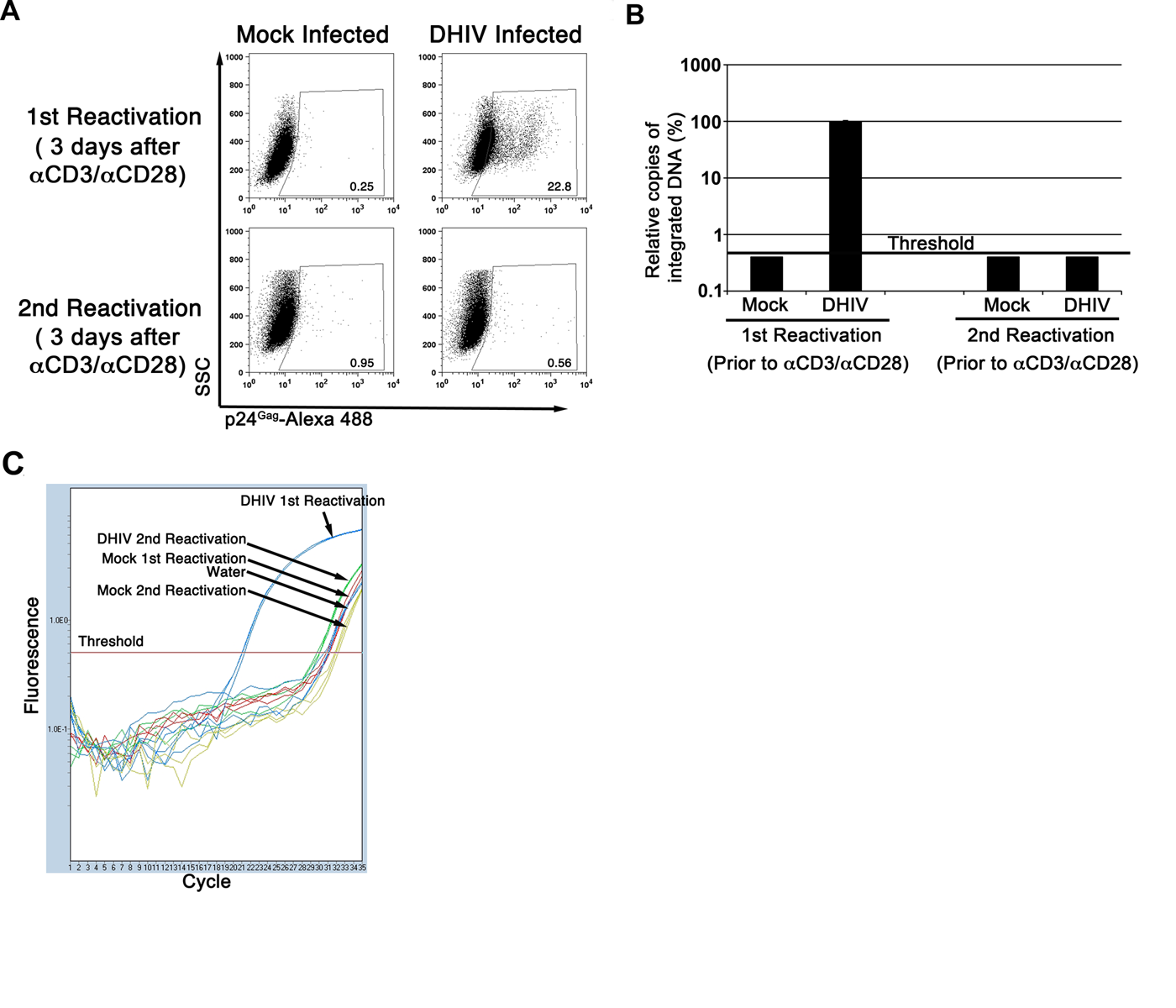

Supplement: Figure S3 — (A) Levels of p24Gag positive cells after reactivation with αCD3/αCD28 antibodies in the 1st and 2nd reactivation for a representative donor. (B) Integrated HIV-1 DNA was analyzed by Alu-LTR PCR in triplicates in the donor from panel A prior to reactivation. Results were normalized relative to the levels of integration in DHIV infected cells before the first reactivation. Mock infected cells and DHIV infected cells prior to second reactivation retrieved a value below the threshold of detection of the technique (C) Amplification curves form the same samples used for B. (TIF) [file ppat.1002288.s003.tif]

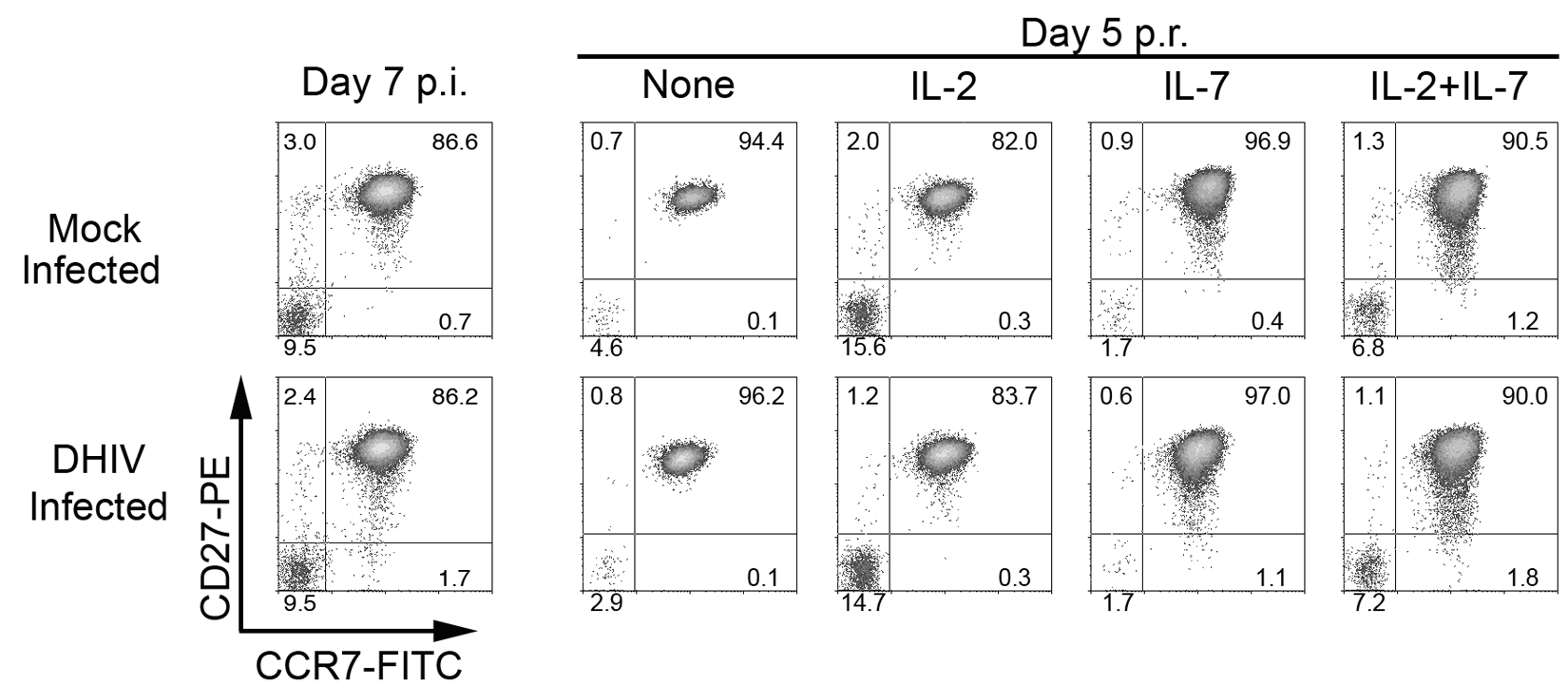

Supplement: Figure S4 — Expression of CCR7 and CD27 in mock or DHIV-latently infected cultured TCM cells at day 14 (Day 7 p.i.). The expression was also monitored 5 days after incubation of the cells in the absence of cytokines (none) or with IL-2, IL-7 or a combination of IL-2 and IL-7 (IL-2+IL-7). (TIF) [file ppat.1002288.s004.tif]
